# Urinary Podocyte Excretion Predicts Urinary Protein Selectivity and Renal Prognosis

**DOI:** 10.1155/2022/2702651

**Published:** 2022-06-29

**Authors:** Makoto Abe, Akiko Kaiga, Takehiro Ohira, Toshihiko Ishimitsu, Akihiro Tojo

**Affiliations:** Department of Nephrology & Hypertension, Dokkyo Medical University, Mibu, Tochigi 321-0293, Japan

## Abstract

**Background:**

Urinary podocyte excretion is related to a reduction in glomerular podocyte numbers, glomerulosclerosis, and urinary protein selectivity. To elucidate the role of urinary podocytes in proteinuria and renal prognosis and to identify the factors that cause podocyte detachment, we examined urinary podocytes in 120 renal biopsy patients.

**Methods:**

Podocytes were identified in urinary sediments stained with fluorescent-labeled anti-podocalyxin antibodies in ten high power fields. The amounts of protein bands, separated by SDS-polyacrylamide gel electrophoresis, were calculated using an image software program and the correlation with urinary podocytes was analyzed. Podocyte surface pores were observed using a low-vacuum scanning electron microscope. The renal prognosis, including induction of hemodialysis or 30% reduction in eGFR, was investigated.

**Results:**

Urinary podocyte excretion showed a higher positive correlation with albumin excretion compared to IgG, prealbumin, and transferrin. There were no significant correlations between urinary podocyte count and low molecular weight proteins, including *β*2-microglobulin and *α*1-microglobulin. The number of podocyte surface pores was positively correlated with proteinuria, suggesting enhanced albumin transcytosis. The hemodynamic pressure on the glomerular capillary wall, including products of pulse pressure and pulse rate (water hammer pressure), was positively correlated with urinary podocyte excretion. Urinary podocyte excretion and Tamm–Horsfall protein (THP) were independent risk factors for renal prognosis but were not related to response to treatment.

**Conclusion:**

Urinary podocyte excretion was correlated with urinary albumin excretion, indicating specific albumin transport by podocytes. Podocytes were detached from the glomerular capillaries by water hammer pressure and THP was involved in the renal prognosis.

## 1. Introduction

Proteinuria is prevented by the glomerular filtration barrier, which is composed of the negative charge of the endothelial glycocalyx and the heparan sulfate of the glomerular basement membrane (GBM) (charge barrier), type IV collagen and laminin (coase-size barrier), and the podocyte slit membrane (fine-size barrier) [1,2]. In various glomerular diseases, podocyte detachment causes an increase in nonselective proteinuria [3]. When podocytes detach, glomerular adhesion lesions develop between the glomerular tuft and Bowman's capsule, gradually progressing to glomerulosclerosis and impairing renal function [4,5]. In fact, we have previously shown that there is a negative correlation between glomerulosclerosis and the number of glomerular podocalyxin-positive podocytes in human renal biopsy specimens [6]. In our previous study, urinary podocytes were found to have a positive correlation with urinary protein excretion, but the correlation with renal damage was not significant, with a small number of 59 cases [6]. It has been reported that urinary podocyte excretion was increased in focal segmental glomerulosclerosis [7,8], IgA nephropathy [9], diabetic nephropathy [10], and postrenal transplantation, and it reflects renal disease activity and progression [11,12]. Thus, in this study, we quantitatively evaluated the number of urinary podocytes shed in 120 renal biopsy patients, observed their renal prognoses, and investigated the role of podocytes in urinary protein selectivity and the factors causing podocyte detachment.

## 2. Materials and Methods

### 2.1. Patients and Clinical Evaluation

This study was reviewed and approved by the Clinical Research Ethics Committee of Dokkyo Medical University (No. R-2-1) and conducted in accordance with the Helsinki Declaration and Dokkyo Medical University Clinical Research Guidelines. We conducted a study of 120 patients who underwent renal biopsy in our department from September 2017 to December 2019.

Blood and urine tests were performed the day before the renal biopsy and the size and resistive index (RI) of the kidney were measured by ultrasonography. The average morning blood pressure and pulse measurements during 4 days of hospitalization for renal biopsy were adopted as the patient's blood pressure and pulse, respectively.  Table 1 shows an analysis of the clinical data at the time of the renal biopsy. Each patient was treated appropriately according to the results of a renal biopsy, and serum creatinine levels and urinary protein were measured at least every 4 months. At the end of July 2021, we analyzed renal prognoses with the introduction of hemodialysis or *a* ≥30% reduction in eGFR as endpoints.

### 2.2. Measurement of Urinary Podocyte Excretion

Early morning first urine was collected on the day of renal biopsy. The urine samples were centrifuged at 500 *g* for 5 minutes, and after removing the supernatant, the sediments with urinary podocytes in 200 *μ*L were stained with phycoerythrin-conjugated monoclonal antibody against human podocalyxin (R&S Systems, Minneapolis, MN, USA) for 30 minutes [6]. We performed double imaging of 577 nm fluorescence and bright field using a BZ-9000 all-in-one fluorescence microscope (KEYENCE Co., Osaka, Japan) to confirm the number of podocytes. The average number of podocytes in 10 visual fields was defined as the number of podocytes excreted in urine.

### 2.3. Measurement of the Urinary Protein Fraction

For the analysis of the urinary protein fraction, 2.5 *μ*L of urine was added to 2.5 *μ*L of sample buffer and 5 *μ*L of distilled water, and 10 *μ*L of the sample was applied to a 4–10% gel and separated by SDS-PAGE. Densitometry was performed using the ImageJ software program (NIH, Bethesda, MD, USA), and the relative concentration of the protein fraction was determined from each band density and area using a standard known concentration of albumin solution [13,14].

### 2.4. Observation of the Glomerular Podocyte Surface by Using a Low-Vacuum Scanning Electron Microscope (LVSEM)

Periodic acid methenamine silver (PAM)-stained 5 *μ*m sections of renal biopsy samples were counterstained with Ponceau-S solution (Olympus Optical Co. LTD., Osaka, Japan) for 15 minutes and observed at 5,000× magnification with an LVSEM (TM4000Plus, Hitachi, Tokyo, Japan). Five to 10 photos of the podocyte surface were taken in each case and the average number of pores in the podocytes was expressed as the pore count per unit area of podocyte surface measured by Image-Pro Plus 5.0 software (Media Cybernetics, Inc., Silver Spring, MD, USA). We investigated the correlation between the number of pores on the podocyte surface and the amount of urinary protein excreted.

### 2.5. Statistics

Data are expressed as mean ± SD. The correlation coefficient was determined between the number of urinary podocytes and urinary blood pressure, pulse, protein, blood data, protein selectivity, and renal echo RI test. A Cox biohazard analysis using the reduction method was performed to identify independent risk factors for the renal prognosis. When the therapeutic responsiveness of urinary protein was compared among the three groups, groups that showed a normal distribution were analyzed by a one-way analysis of variance followed by the Bonferroni posttest to compare between groups. A Kruskal–Wallis nonparametric analysis followed by Steel–Dwass multiplex comparison was performed to for the analysis of nonnormally distributed urinary podocytes, urinary proteins, RI, and other parameters. Complete remission (CR) was defined with proteinuria less than 0.3 g/gCr, partial remission (PR) with proteinuria less than 3.5 g/gCr, and nephrotic syndrome (NS) remained proteinuria more than 3.5 g/gCr, and the percent usage of therapeutic drugs among CR, PR, and NS was analyzed by chi-square test. Statistical analyses were performed using SSRI software version 1.02 (Social Survey Research Information Co., Ltd., Tokyo, Japan), and SPSS software version 28.0.0.0 (IBM, Chicago, USA), and *p* values of <0.05 were considered statistically significant.

## 3. Results

### 3.1. Urinary Podocyte and Protein Fraction

**Figure 1:**
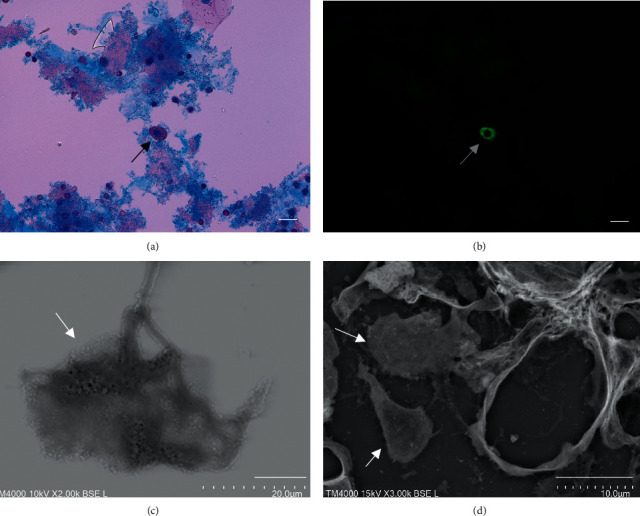
Light microscopy of urinary podocytes (a), immunofluorescence staining for podocalyxin (b), LVSEM observation (c), and PAM staining of the renal biopsy sample of the same patient (d). The arrows indicate podocytes. Bars indicate 10 *µ*m.

The urinary podocytes were confirmed based on observation with light microscopy (Figure 1(a)) and fluorescence microscopy for the podocalyxin antibody (Figure 1(b)) at the same time and in the same place using an all-in-one microscope. LVSEM observations of the podocyte showed many small exocytosis holes on the cell surface with primary processes (Figure 1(c)). The LVSEM observation of the renal biopsy section in the same patient showed a podocyte detached from the capillary wall (Figure 1(d)). Other urinary cells, such as squamous cells, tubular epithelial cells, and white blood cells, were not stained by the podocalyxin antibody (Supplementary Figure 1).

**Figure 2:**
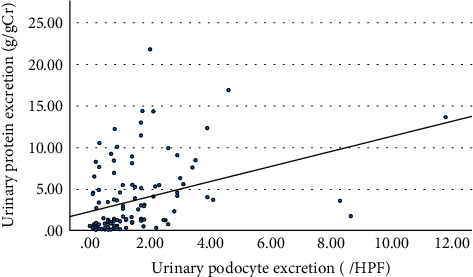
Relationship between urinary podocyte excretion and the amount of proteinuria. r = 0.38, *p* < 0.001 (*n* = 120).

The number of podocytes excreted in urine was expressed as the average number of cells labeled with podocalyxin in 10 high-magnification fields and was significantly correlated with urinary protein excretion (Figure 2).

**Figure 3:**
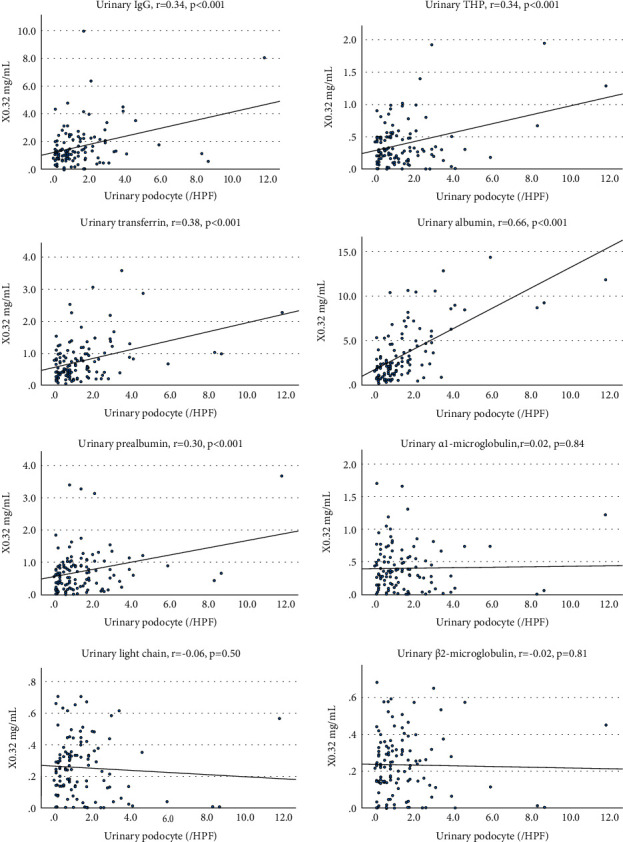
The correlation between urinary podocyte excretion and urinary protein fractions. The r and *p* values were as follows: urinary podocytes and IgG, *r* = 0.34, *p* < 0.001; Tamm–Horsfall protein (THP), *r* = 0.34, *p* < 0.001; transferrin (Tf), *r* = 0.38, *p* < 0.001; albumin, *r* = 0.66, *p* < 0.001; prealbumin, *r* = 0.30, *p* < 0.001; *α*1-microglobulin, *r* = 0.02, *p*=0.84; light chain (LC), *r* = −0.06, *p*=0.50; and *β*2-microglobulin, *r* = −0.02, *p*=0.81 (*n* = 120).

The correlation coefficient between the urinary podocyte excretion and urinary protein fraction, as analyzed by SDS-PAGE (Supple Figure 2), showed the best correlation with urinary albumin excretion (*r* = 0.66, *p* < 0.001), followed by significance with transferrin (*r* = 0.38, *p* < 0.001) (Figure 3). When podocytes were shed, nonselective proteinuria was formed and high molecular weight IgG and THP were easily excreted in the urine, resulting in a significant correlation; however, the correlation coefficient was lower than that of albumin. On the other hand, no correlation was found between urinary podocytes and small molecular weight proteins of *α*1-microglobulin, light chain, and *β*2-microglobulin (Figure 3).

### 3.2. Podocyte Surface Pores by LVSEM and Proteinuria

**Figure 4:**
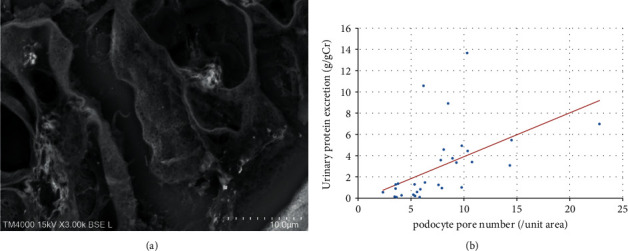
LVSEM observation of podocyte surface pores (a) and the correlation between podocyte pore number and urinary protein excretion (b). r = 0.52, *p* < 0.005 (*n* = 31).

Observations by LVSEM showed many small pores in the apical membranes of podocytes (Figure 4(a)), and the number of small pores showed a positive correlation with urinary protein excretion (Figure 4(b)). The LVSEM observation of the glomeruli form tubulointerstitial nephritis showed a normal foot process with few pores (Supplementary Figure 3).

### 3.3. Podocyte Detachment and Water Hammer Pressure

**Figure 5:**
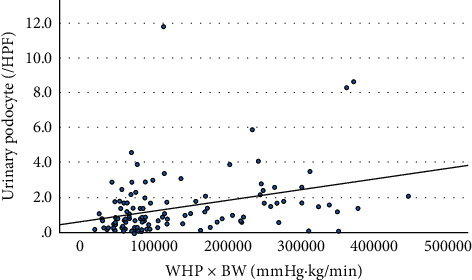
Correlation between water hammer pressure × BW and urinary podocyte excretion. r = 0.35, *p* < 0.001 (*n* = 120).

To clarify the factors involved in urinary podocyte excretion, Table 2 shows the correlation with clinical data obtained at the time of renal biopsy. The number of urinary podocytes showed a positive correlation with urinary protein excretion and NAG and a negative correlation with serum total protein and albumin. These correlations were interpreted as a result of the loss of podocytes. On the other hand, as the factors involved in podocyte shedding, the number of urinary podocytes showed a significant positive correlation with pulse pressure (PP) × pulse rate (PR), that is, water hammer pressure (WHP). In particular, the product of WHP × body weight showed the better correlation (Figure 5). This indicates that the physical force in the glomerular capillaries is important for podocyte shedding.

### 3.4. Podocyte Detachment and Renal Prognosis

We investigated factors of renal prognosis. Age, sex, urinary podocyte, mean of systolic and diastolic blood pressure during 4 days in the hospital, serum creatinine, serum IgG, urinary protein, urinary Tamm–Horsfall protein (THP), and resistive index (RI) at the time of renal biopsy were significant risk factors for renal prognosis, including dialysis induction, or 30% reduction in eGFR, by the Cox biohazard analysis (Table 3). Increased urinary excretion of THP will cause cast formation and obstruct nephron flow, and increased RI will contribute to the reduction of renal blood flow. However, urinary podocyte excretion showed good prognosis for renal prognosis (HR 0.29, *p* < 0.007), suggesting that the patients undergoing renal biopsy were cases with acute active nephritis or nephrotic syndrome with increased podocyte excretion and those cases often show a good response to treatment.

**Figure 6:**
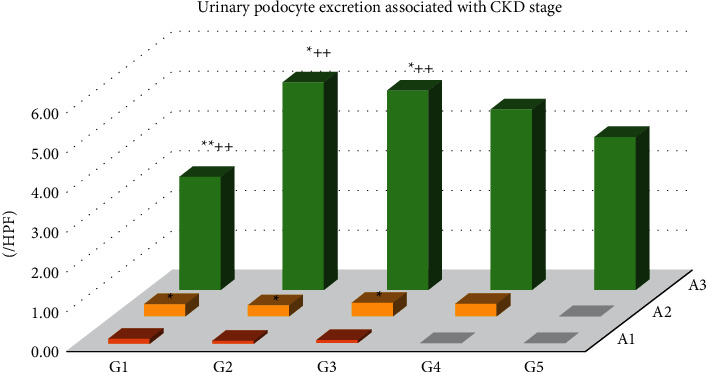
Urinary podocyte excretion associated with CKD stages with G1 to G5 as well as with A1 to A3. ^*∗*^*p* < 0.05 and ^*∗∗*^*p* < 0.01 vs. A1, ^++^*p* < 0.01 vs. A2.

The number of podocytes in urine was lower in the early unactive cases with proteinuria A1 and A2 (<0.5 g/gCr) than those with A3 (≥0.5 g/gCr) in stages G1-G3 of CKD and was also lower in stage G1, as well as in the chronic advanced stage G5 (Figure 6). Urinary podocyte number increased in the active cases with proteinuria A3 in G2 and G3 stage (Figure 6) that will show good response to the treatment (Supplementary Figures 4 and 5). The urinary podocyte is better to express as mean of 10 high-power fields' (HPF) count than those corrected by urinary creatinine, as urinary creatinine excretion decreased in some cases with reduced medullary concentrating function, which cause overestimation of urinary podocyte (Supplementary Figure 4).

### 3.5. Clinical Factors Affecting Therapeutic Responsiveness of Urinary Protein

We examined clinical data at the time of renal biopsy that affected the response of urinary protein amount at the final observation (Table 4). At the time of renal biopsy, serum creatinine, proteinuria, and NAG were significantly lower in the CR group than in the NS group. A mean RI of 0.64 on renal Doppler ultrasonography resulted in the CR or PR groups, while a mean RI of 0.72 remaining in the NS group. Interestingly, the WHP was significantly lower in the CR group. The percentage of therapeutic agents used during observation is summarized in Table 4. After renal biopsy, most cases with nephrotic syndrome and active nephritis were treated with steroid/immunosuppressant, which was used significantly more in the CR group. On the other hand, chronic cases with glomerulosclerosis and interstitial fibrosis were treated with angiotensin converting enzyme inhibitor (ACEI)/angiotensin receptor blocker (ARB) or calcium channel blocker (CCB), which were used significantly more in the NS group (Table 4).

## 4. Discussion

This study revealed that podocyte shedding in urine was most strongly correlated with albuminuria by SDS-PAGE urinary protein fractions and that the observation of increased podocyte pores under an LVSEM was correlated with proteinuria in human nephrotic syndrome. Furthermore, it was clarified that the WHP on the glomerular capillary wall was important for the shedding of podocytes in urine. Urinary THP excretion was also an independent risk factor for the renal prognosis, in addition to previously known risk factors such as serum creatinine, proteinuria, and RI.

In this study, the number of podocytes shed in urine was correlated with urinary protein excretion (Figure 1), which was in line with the results of a previous study [6]. It has been newly discovered that urinary podocyte excretion was most strongly correlated with albumin in the urinary protein fraction and affects urinary protein selectivity. On the other hand, the correlation with high molecular weight proteins (IgG and THP) was weak, suggesting that loss of podocytes reduced the selectivity of urinary proteins. Surprisingly, no significant correlation was shown with small molecular weight proteins, such as LC, *α*1MG, and *β*2MG. This cannot be explained by the theory of slit membranes because abnormalities in the slit membrane cause filtration of albuminuria and low molecular proteins from enlarged slit pores [3]. Until now, selective albuminuria has generally been thought to selectively filter albumin due to impairment of the basement membrane charge barrier [15] or abnormalities in the slit membrane size barrier [16–18].

In PAN nephrotic rats, the expression of FcRn is increased in podocytes, which selectively transcytoses albumin as a receptor for albumin [3,13]. Motor proteins such as cytoplasmic dynein-1, myosin-9, and myosin-7, which are involved in podocyte vesicle transport, are increased in the glomerulus of the PAN nephrotic syndrome model [14]. In this human study, the number of small holes created by transcytosis of the apical membrane of podocytes showed a significant correlation with urinary protein excretion (Figure 4). When foot process effacement was observed in nephritis or nephrotic syndrome, vesicular transport of podocytes was enhanced in association with an increase in urinary proteins, especially albuminuria. This supports the theory that selective albuminuria is exhibited by albumin transcytosis in podocytes [3].

Animal models of focal glomerulosclerosis show that hemodynamic forces, integrins, cell cycle, oxidative stress, advanced glycated end-products, and other factors are involved in the shedding of podocytes in urine [19,20]. In this human study, the factors that were correlated with the number of podocytes in urine are shown in Table 2, and the decrease in serum total protein, serum albumin, and renal length is believed to be the result of podocyte shedding. However, SBP, PP, and PR are physical forces acting on the glomerular capillary wall, of which PP × PR provides WHP which is important for podocyte shedding from the basement membrane, and WHP × BW was closely correlated with urinary podocyte excretion (Figure 5). The number of glomerular podocytes decreases due to aging and obesity and causes glomerulosclerosis [21–23]. The shedding of urinary podocytes was also associated with urinary protein, urinary WBC, NAG, and SI (Table 2). We have previously reported that urinary podocyte excretion did not correlate with the glomerulosclerosis score (*r* = 0.01, *p*=0.95), but showed a significant negative correlation with the number of glomerular podocytes (*r* = −0.43, *p* < 0.01) [6]. Especially in patients with minimal change nephrotic syndrome and focal segmental glomerulosclerosis, glomerular and urinary podocytes are associated with decreased proteinuria selectivity [6]. In clinical practice, ACEI/ARBs are often used to reduce intraglomerular pressure and urinary protein to prevent glomerular damage [23–25]. In addition to BW control, both the lowering of the glomerular pressure with ACEI/ARBs and regulation of pulse rate with *β*-blockers may be useful for reducing WHP and preventing podocyte shedding and suppressing FSGS. As the percentage of therapeutic agents was merely selected by the clinical and histological status (Table 4) and we did not measure urinary podocyte after treatment, therefore, we could not know the therapeutic effects on the urinary podocytes. Further study is necessary to elucidate therapeutic effect of these drugs on podocyte shedding.

In IgA nephropathy, it is reported that an increase in urinary podocyte shedding decreases the number of glomerular podocytes and is correlated with renal tissue damage; thus, it may be an indicator of the renal prognosis [9,12]. However, it has been found that urinary podocytes in renal biopsy patients show a biphasic change that increases in the acute phase and decreases in the chronic phase [6], and in the present study we confirmed the changes in urinary podocyte excretion associated with CKD stage in Figure 6 and Supplementary Figure 5. In the present study, a hazard ration of urinary podocyte excretion for the renal endpoint was 0.29 (*p* < 0.01, Table 3). Patients with less urinary podocyte excretion showed a good renal prognosis after renal biopsy. This indicates that cases with fewer podocyte excretion include both early-phase renal disease and advanced glomerular disease with fewer podocytes remaining in the glomeruli. Moreover, active cases with increased urinary podocyte excretion were effectively treated and showed good prognosis during short-time observation. Further long-time observation is necessary for the effect of urinary podocytes in the renal prognosis. Interestingly, the Tamm–Horsfall protein (THP) secreted from the thick ascending limb of the loop of Henle was a significant exacerbating factor, with a hazard ratio of 36.66 (*p* < 0.01, Table 3). THP is a protein that is the source of hyaline casts that are observed in physiological conditions, including dehydration, the use of diuretics, and exercise, and has been thought to have little pathological significance [26,27]. Our results in this study clarified that hyaline casts, which block the nephron tubular flow, are independent risk factors for the renal prognoses. Recently, the THP protein was identified as a uromodulin, which has been shown to regulate NKCC2 expression in the loop of Henle and to be involved in the development and exacerbation of hypertension [28]. This study shows that THP is an important target for the treatment of nephritis and nephrotic syndrome. There are clinical studies showing that treatment with sodium bicarbonate improves the renal function in CKD patients because the alkalization of urine with sodium bicarbonate suppresses the formation of hyaline casts [29]. Further research is needed to elucidate the effect of sodium bicarbonate on the suppression of THP and the improvement of renal prognosis in a prospective, randomized controlled trial.

As shown in Table 4, both WHP and RI on renal biopsy were significantly lower in patients who achieved complete remission with treatment. We have previously shown that steroid treatment is effective in cases of RI 0.65–0.70 [6]. In this study, the RI in the complete remission group was 0.64 ± 0.01, consistent with previous results. It was newly discovered that a lower WHP is important for response to treatment. Unfortunately, the number of podocytes in the urine did not affect the response to treatment. This is because active nephritis increases the number of podocytes in the urine [11,12], but decreases the number of podocytes in the urine of mild early and advanced chronic cases [6].

### 4.1. Limitations

There are several limitations of this study. There are many factors for podocyte detachment from glomerular capillaries, other than water hammer pressure. We could not investigate podocyte apoptosis, oxidative stress, drug influence, and so on. We have previously revealed the importance of oxidative stress in podocytes and the protective effect of ARB in our previous experimental studies [13,30,31]. Urinary podocytes have been reported to be viable and half of them showed increased apoptosis signaling [32]. Further clinical studies are necessary to clarify the mechanism of podocyte detachment. In addition, since we could not evaluate urinary podocytes after treatment, it is not possible to clarify the effect of therapeutic drug on podocyte detachment.

## 5. Conclusions

Urinary podocytes were closely correlated with albuminuria in association with an increase in exocytosis pits on the surface of podocytes, suggesting selective albumin transport by podocytes. WHP as a physical force on glomerular capillaries correlated with urinary podocytes, and urinary THP excretion was associated with renal prognosis.

## Figures and Tables

**Table 1:** Clinical data at the time of renal biopsy.

Clinical background data	Mean ± standard deviation
Age	50.0 ± 19.5
Sex: male : female	56 : 64
Blood pressure at day of admission (mmHg)	131 ± 18/77 ± 12
Blood pressure 4-day mean during hospitalization (mmHg)	103 ± 21/75 ± 10
Pulse rate 4-day mean (beats/min)	74 ± 9
Serum creatinine (mg/dl)	1.41 ± 1.39
eGFR (mL/min/1.73 m^2^)	61.5 ± 32.5
Serum total protein (g/dL)	6.1 ± 1.3
Serum albumin (g/dL)	3.0 ± 1.1
Serum IgG (mg/dL)	1206 ± 683
Urinary protein (g/gCr)	3.54 ± 4.22
Urinary red blood cells (/HPF)	30 ± 34
Selectivity index	0.298 ± 0.267
Long axis of renal size (cm)	10.1 ± 1.2
Short axis of renal size (cm)	4.8 ± 0.7
Resistive index	0.65 ± 0.09
Histological diagnosis	
IgA nephropathy/vasculitis	33 cases
Membranous nephropathy	15 cases
ANCA vasculitis	14 cases
Minimal change nephrotic syndrome	13 cases
Tubulointerstitial nephritis	12 cases
Focal segmental glomerulosclerosis	9 cases
Diabetic nephrosclerosis	8 cases
Lupus nephritis	6 cases
Thin membrane disease/minor glomerular abnormalities	5 cases
Poststreptococcal acute glomerulonephritis	2 cases
Membranoproliferative glomerulonephritis	1 case
Non-IgA nephropathy	1 case
C3 nephropathy	1 case

**Table 2:** Correlation of clinical parameters with urinary podocytes.

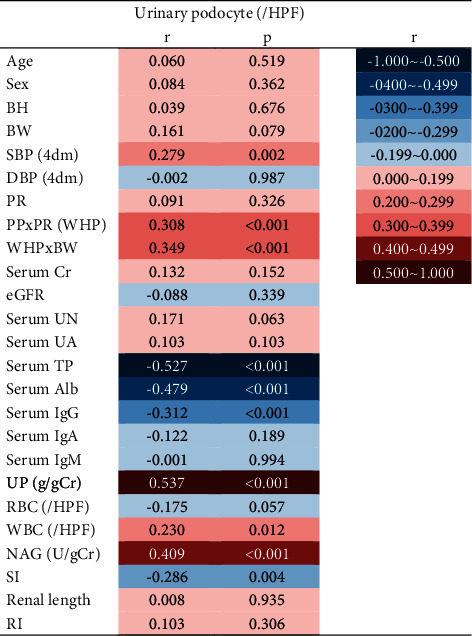

BW indicates body weight; SBP, systolic blood pressure; PP, pulse pressure; PR, pulse rate; WHP, water hammer pressure; UP, urinary protein; NAG, N-acetyl-*β*-D-glucosaminidase; Tf, transferrin; MG, microglobulin; SI, selectivity index; RI, resistive index.

**Table 3:** Possible risk factors for renal prognosis, including HD or eGFR-30% reduction, by multivariate analysis using a Cox proportional hazards model.

	Hazard ratio	95% CI	*p* value
Age	0.93	0.88–0.98	0.007
Sex (m 1/f 0)	0.16	0.03–0.74	0.019
Urinary podocyte	0.29	0.12–0.71	0.007
BW	1.08	1.01–1.12	0.051
SBP (4ds' mean)	1.06	1.00–1.13	0.025
DBP (4ds' mean)	0.85	0.75–0.96	0.012
Serum Cr	1.77	1.13–2.77	0.013
Serum IgG	1.001	1.001–1.003	0.004
Urinary protein	1.49	1.10–2.01	0.009
THP	36.66	2.33–576.59	0.010
RI	2470102	150–40735861555	0.003

BW indicates body weight; SBP, systolic blood pressure; DBP, diastolic blood pressure; Cr, creatinine; THP, Tamm–Horsfall protein; RI, resistive index.

**Table 4:** Physiological data at the time of the renal biopsy according to the response of proteinuria.

	CR	PR	NS	*P*
Age (year-old)	48 ± 2	52 ± 3	58 ± 4	0.27
Sex (M1:F0)	0.44 ± 0.06	0.55 ± 0.08	0.33 ± 0.17	0.37
BH (cm)	160.4 ± 1.1	162.2 ± 1.3	158.0 ± 4.5	0.44
BW (kg)	62.5 ± 1.6	64.9 ± 2.1	57.8 ± 5.6	0.34
SBP (mmHg)	101 ± 2	104 ± 3	118 ± 8	0.07
DBP (mmHg)	75 ± 1	76 ± 1	75 ± 4	0.89
PR (/min)	73 ± 1	74 ± 2	75 ± 4	0.08
PP × PR (WHP) (mmHg/min)	1910 ± 144	2126 ± 262	3382 ± 594^*∗∗*^^+^	0.014
WHP × BW (mmHg kg/min)	122060 ± 10764	136609 ± 17076	185601 ± 28861	0.07
Serum Cr (mg/dL)	1.17 ± 0.14	1.81 ± 0.28^*∗*^	1.63 ± 0.39	0.018
eGFR (ml/min/1.73m2)	67.3 ± 3.56	54.9 ± 5.82	43.0 ± 7.60^*∗*^	0.023
Serum UN (mg/dL)	22.1 ± 1.80	24.1 ± 2.10	26.1 ± 3.50	0.64
Serum UA (mg/dL)	6.6 ± 0.95	6.1 ± 0.25	6.4 ± 0.54	0.93
Serum TP (g/dL)	6.0 ± 0.16	6.3 ± 0.17	6.1 ± 0.44	0.56
Serum Alb (g/dL)	3.0 ± 0.15	3.0 ± 0.12	2.6 ± 0.20	0.56
Serum IgG (mg/dL)	1108 ± 63.5	1339 ± 126	1445 ± 392	0.14
UP (g/gCr)	3.28 ± 0.49	3.21 ± 0.65	7.06 ± 1.32^*∗∗*^^++^	0.004
RBC (/HPF)	33.4 ± 4.2	28.3 ± 5.2	13.4 ± 5.2	0.22
WBC (/HPF)	9.9 ± 1.4	11.1 ± 3.2	9.7 ± 2.9	0.91
NAG (U/gCr)	19.3 ± 2.53	21.1 ± 3.25	42.8 ± 11.1^*∗*^^+^	0.010
Urinary IgG	1.49 ± 0.15	1.74 ± 0.29	2.43 ± 0.55	0.17
Urinary THP	0.39 ± 0.05	0.38 ± 0.05	0.36 ± 0.08	0.96
Urinary Tf	0.77 ± 0.08	0.74 ± 0.09	0.79 ± 0.12	0.95
Urinary albumin	3.07 ± 0.35	3.60 ± 0.52	5.02 ± 1.02	0.17
Urinary prealbumin	0.73 ± 0.08	0.67 ± 0.07	0.94 ± 0.30	0.52
Urinary *α*1MG	0.37 ± 0.03	0.48 ± 0.07	0.38 ± 0.10	0.26
Urinary LC	0.17 ± 0.20	0.20 ± 0.22	0.20 ± 0.06	0.37
Urinary *β*2MG	0.16 ± 0.18	0.18 ± 0.20	0.24 ± 0.06	0.52
SI	0.31 ± 0.04	0.29 ± 0.03	0.27 ± 0.04	0.92
Urinary podocyte (/HPF)	1.41 ± 0.24	1.41 ± 0.18	1.70 ± 0.45	0.14
Renal length (cm)	10.3 ± 0.12	10.4 ± 0.22	10.4 ± 0.48	0.28
RI	0.64 ± 0.01	0.64 ± 0.02	0.72 ± 0.02^*∗*^	0.046
Steroid/immunosuppressant	77%	66%	22%^*∗∗*^^+^	0.0033
ARB/ACEI	52%	68%	89%	0.046
*β*-Blocker	11%	13%	11%	0.94
CCB	32%	63%^*∗∗*^	78%^*∗*^	0.001
MR blocker	8%	29%^*∗*^	11%	0.0140
Diuretics	36%	45%	56%	0.397

CR, complete remission; PR, partial remission; NS, nephrotic syndrome; WHP, water hammer pressure; NAG, N-acetyl-*β*-D-glucosaminidase; THP, Tamm–Horsfall protein; Tf, transferrin; MG, microglobulin; SI, selectivity index; RI, resistive index; CCB, calcium channel blocker; MR, mineral corticoid receptor. ^*∗*^*p* < 0.05 and ^*∗∗*^*p* < 0.01 vs. CR; ^+^*p* < 0.05 and ^++^*p* < 0.01 vs. PR.

## Data Availability

The data used to support the findings of this study are available from the corresponding author upon request.

## Supplementary Materials

Supplementary Materials Urine in the normal control (Supplementary Figure 1), representative SDS-PAGE gel of urinary proteins (Supplementary Figure 2), LVSEM observation of normal glomerulus (Supplementary Figure 3), comparison of data expressed the podocyte number by/HPF and by/gCr (Supplementary Figure 4), and comparison of urinary podocyte number in accordance with GFR grades and urinary protein levels associated with renal endpoint and treatment responsiveness (Supplementary Figure 5). Supplementary Figure 1: urinary sediment (a) and immunofluorescence staining for podocalyxin (b) in the urine of normal control. Bars indicate 10 *µ*m. Squamous cell, tubular epithelial cell, and uromucoid were not stained by podocalyxin antibodies. Supplementary Figure 2: representative SDS-PAGE gel of urinary proteins from patients with IgA nephropathy (lanes 1, 2), tubulointerstitial nephritis (lanes 3, 14), ANCA-related crescentic glomerulonephritis (lane 4), membranous nephropathy (lane 5), focal segmental glomerulosclerosis (lane 6), diabetic nephrosclerosis (lanes 7, 9), minor glomerular abnormalities (lane 8), C3 nephropathy (lane 10), minimal change nephrotic syndrome (lane 11), and IgA vasculitis (lanes 12, 13). Supplementary Figure 3: LVSEM observation of podocytes from tubulointerstitial nephritis. Podocytes with normal foot processes are observed (arrows). Supplementary Figure 4: distribution of data showing urinary podocytes per high-power field (a) or urinary podocytes corrected by urinary creatinine (b) with final serum creatinine. Black circles indicate patients with renal endpoint and white circles those with renal survival. Urinary podocyte in the first urine on the day of renal biopsy expressed podocyte/HPF showed better distribution than those corrected for by urinary creatinine. Supplementary Figure 5: comparison of urinary podocyte excretion between the patients with renal survival and renal endpoint in renal prognosis (a, c) or between patients with complete remission (CR) and non-CR in response to treatment (b, d). The patients were classified with estimated glomerular filtration rate (eGFR) of CKD stages of G1 to G5 (a, b) or with urinary protein at the time of renal biopsy (c, d). G1, eGFR ≥90 mL/min/1.73 m^2^; G2, 60–89; G3, 30–59, G4, 15–29; G5, <15. UP1, urinary protein <0.5 g/gCr; UP2, 0.5–3.4; UP3, ≥3.5. ^*∗*^*p* < 0.05, ^*∗∗*^*p* < 0.01. Urinary podocyte excretion was significantly more in the patients who reached renal endpoint in the G2 stage, whereas it is not increased in the cases with the early G1 stage and advanced G5 stage. Urinary podocyte excretion increased with increasing urinary protein, but there were no significant differences between cases with renal survival and renal endpoint. Urinary podocyte excretion was significantly less in patients with CR compared to those without CR at stage G1, while it was significantly more in CR cases than in non-CR cases at stage G4. Patients who reached CR after treatment showed significantly more urinary podocytes associated with increased urinary protein.
