# Supplementary material for: Urinary Podocyte Excretion Predicts Urinary Protein Selectivity and Renal Prognosis
Source: Int J Nephrol. 2022 Jun 29;2022:2702651. doi: 10.1155/2022/2702651 (PMC9296344; doi:10.1155/2022/2702651)
Supplement: Supplementary Materials — Urine in the normal control (Supplementary Figure 1), representative SDS-PAGE gel of urinary proteins (Supplementary Figure 2), LVSEM observation of normal glomerulus (Supplementary Figure 3), comparison of data expressed the podocyte number by/HPF and by/gCr (Supplementary Figure 4), and comparison of urinary podocyte number in accordance with GFR grades and urinary protein levels associated with renal endpoint and treatment responsiveness (Supplementary Figure 5). Supplementary Figure 1: urinary sediment (a) and immunofluorescence staining for podocalyxin (b) in the urine of normal control. Bars indicate 10 µm. Squamous cell, tubular epithelial cell, and uromucoid were not stained by podocalyxin antibodies. Supplementary Figure 2: representative SDS-PAGE gel of urinary proteins from patients with IgA nephropathy (lanes 1, 2), tubulointerstitial nephritis (lanes 3, 14), ANCA-related crescentic glomerulonephritis (lane 4), membranous nephropathy (lane 5), focal segmental glomerulosclerosis (lane 6), diabetic nephrosclerosis (lanes 7, 9), minor glomerular abnormalities (lane 8), C3 nephropathy (lane 10), minimal change nephrotic syndrome (lane 11), and IgA vasculitis (lanes 12, 13). Supplementary Figure 3: LVSEM observation of podocytes from tubulointerstitial nephritis. Podocytes with normal foot processes are observed (arrows). Supplementary Figure 4: distribution of data showing urinary podocytes per high-power field (a) or urinary podocytes corrected by urinary creatinine (b) with final serum creatinine. Black circles indicate patients with renal endpoint and white circles those with renal survival. Urinary podocyte in the first urine on the day of renal biopsy expressed podocyte/HPF showed better distribution than those corrected for by urinary creatinine. Supplementary Figure 5: comparison of urinary podocyte excretion between the patients with renal survival and renal endpoint in renal prognosis (a, c) or between patients with complete remission (CR) and non-C [file 2702651.f1.zip › Supple Figure 4.pptx]

## Slide 1
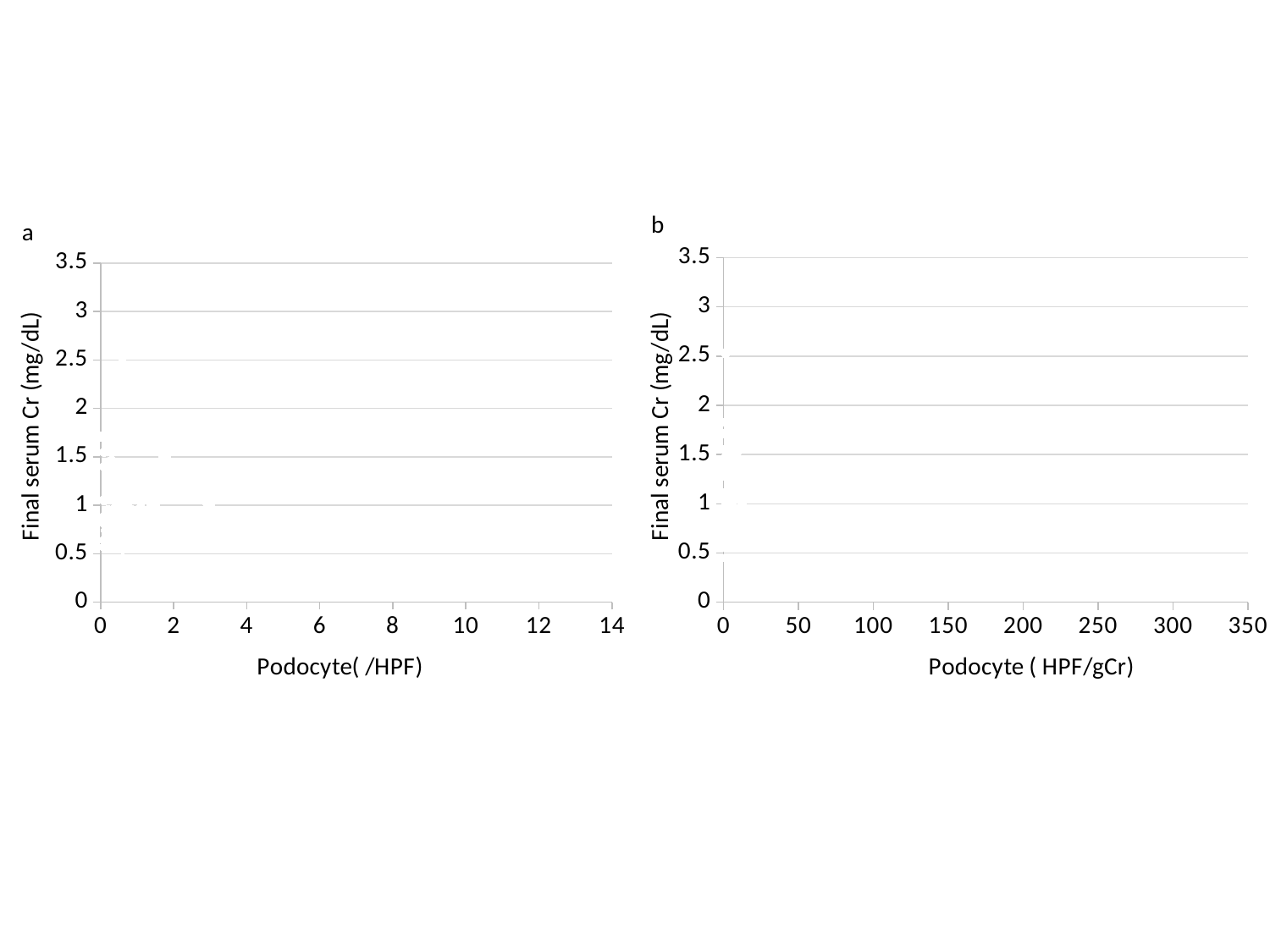

b
a
### Chart
| Category | 最終Cr（腎予後1）　 | 最終Cr（腎予後0）　　　 |
|---|---|---|
### Chart
| Category | 最終Cr（腎予後1）　 | 最終Cr（腎予後0）　　　 |
|---|---|---|Final serum Cr (mg/dL)
Final serum Cr (mg/dL)
